# On the Role of Attention in Binocular Rivalry: Electrophysiological Evidence

**DOI:** 10.1371/journal.pone.0022612

**Published:** 2011-07-22

**Authors:** Urte Roeber, Sandra Veser, Erich Schröger, Robert P. O'Shea

**Affiliations:** 1 Institute for Psychology, University of Leipzig, Leipzig, Germany; 2 Discipline of Psychology, School of Health and Human Sciences, Southern Cross University, Coffs Harbour, Australia; 3 Biomedical Science, The University of Sydney, Sydney, Australia; 4 Institute of Medical Psychology, University of Tübingen, Tübingen, Germany; 5 Department of Psychology, University of Otago, Dunedin, New Zealand; 6 Discipline of Psychology and Cognitive Neuroscience Research Cluster, School of Health and Human Sciences, Southern Cross University, Coffs Harbour, Australia; University of Minnesota, United Statzes of America

## Abstract

During binocular rivalry visual consciousness fluctuates between two dissimilar monocular images. We investigated the role of attention in this phenomenon by comparing event-related potentials (ERPs) when binocular-rivalry stimuli were attended with when they were unattended. Stimuli were dichoptic, orthogonal gratings that yielded binocular rivalry and dioptic, identically oriented gratings that yielded binocular fusion. Events were all possible orthogonal changes in orientation of one or both gratings. We had two attention conditions: In the attend-to-grating condition, participants had to report changes in perceived orientation, focussing their attention on the gratings. In the attend-to-fixation condition participants had to report changes in a central fixation target, taking attention away from the gratings. We found, surprisingly, that attending to rival gratings yielded a smaller ERP component (the N1, from 160–210 ms) than attending to the fixation target. To explain this paradoxical effect of attention, we propose that rivalry occurs in the attend-to-fixation condition (we found an ERP signature of rivalry in the form of a sustained negativity from 210–300 ms) but that the mechanism processing the stimulus changes is more adapted in the attend-to-grating condition than in the attend-to-fixation condition. This is consistent with the theory that adaptation gives rise to changes of visual consciousness during binocular rivalry.

## Introduction

When two different images are presented continuously, one each to the same retinal location of the two eyes, one sees the remarkable changes in visual consciousness known as binocular rivalry, discovered by Porta in 1593 [1]: One sees one of the images for a few moments, referred to as the dominant image, while the other is completely invisible, suppressed. Then, after a brief period of transition, when both or parts of the two images are seen together, the other image becomes dominant and the first becomes suppressed. The images continue to alternate in visual consciousness randomly as long as one bothers to look at them. Binocular rivalry is an important phenomenon for researching the neural correlates of consciousness because visual consciousness changes without any change in the physical stimulation [2].

Binocular rivalry has been explained by two classes of theories: top-down, mechanisms involving attention [3], [4], [5] versus bottom-up mechanisms involving reciprocal inhibition and adaptation [6], [7], [8], [9], [10]. Our aim was to investigate the role of attention in binocular rivalry by tapping into the neural mechanisms using event-related potentials (ERPs), when attention is either on binocular-rivalry stimuli or when it is on some other stimulus.

Originally, attention was considered to be the cause of binocular rivalry [3], [4]. But this notion has been abandoned [11], [12], mainly because of the limited success observers have when they use attention to try to see only one of the rival images or to influence the rate of rivalry [12], [13]. However it is still possible that attention is required for binocular rivalry. This has been investigated by studying the effects of taking attention away from the rival stimuli on perception, on blood flow in the brain, and on the electrical activity of the brain.

To investigate the effects on perception of taking attention away from the rival stimuli, Paffen, Alais, and Verstraten [14] asked their participants either to attend to central rivalry stimuli and to indicate the current dominant rival percept or also to monitor a peripheral display of randomly moving dots for a brief episode of coherent motion. Paffen et al. found that the rivalry-alternation rate is faster when attention is on the rival stimuli than when some attention is taken away from the rival stimuli, showing that attention affects rivalry.

To investigate the effects on blood flow in the brain (using functional magnetic resonance imaging, fMRI) of taking attention away from the rival stimuli, Lee, Blake, and Heeger [15] asked their participants either to attend to the rival stimuli and to indicate the current dominant rival percept or to attend to a difficult task at fixation and not to respond to the rival stimuli at all. They found that fMRI responses are stronger when attention is on the rival stimuli versus when attention is diverted from the rival stimuli. They also found that an fMRI signature of rivalry from V2 is present only when attention is on the rivalry stimuli.

To investigate the effects on electrical activity of the brain of taking attention away from the rival stimuli, Zhang, Engel, Rios, He, and He [16] asked their participants either to attend and respond to rival stimuli or to attend and respond to a difficult task at fixation. They found that a signature of rivalry from frequency-tagged-EEG activity (a negative correlation between activity from the two monocular stimuli) is present when attention is on the rival stimuli, but absent when not. They concluded that attention is necessary for rivalry to occur. There have been other studies in which attention is directed to one of the rival stimuli that have shown enhanced electrical activity associated with the attended stimulus [17], [18]; we will discuss these later.

The behavioural, fMRI, and EEG evidence is consistent with attention's being required for rivalry to occur. But Paffen et al. proposed an intriguing alternative hypothesis, at least for their behavioural results. They proposed that:

- Attention is *not* required for rivalry to occur,
- Attention increases the underlying neural activity of each of the representations of the rival stimuli that compete in the low-level rivalry mechanism; this is similar to increasing the contrast of the rival stimuli, and
- This increase in activity leads to greater adaptation, leading to faster alternations.

Paffen et al. supported this explanation by showing that they could speed up rivalry alternations by the same amount as when attention is on the rival stimuli simply by increasing the contrast of the rival stimuli. It is quite likely that fMRI activity and frequency-tagged EEG activity is also stronger when the underlying neural activity in a low-level mechanism is greater.

We decided to test Paffen et al.'s explanation of attention's effects on rivalry by measuring ERPs. ERPs are changes in electrical activity of the brain that follow some event, measured from electrodes placed on the scalp [19]. ERPs have temporal resolution in the order of milliseconds. The typical form of the ERP when the event is the sudden appearance of a specific visual object or feature includes a positive component peaking about 100 ms after the event, the *P1*, and a negative component about 170 ms after the event, the *N1*.

There is a huge body of evidence from tasks other than rivalry that ERP amplitudes are enhanced for attended visual stimuli or stimulus features as compared to ignored (or to less-attended) visual stimuli or stimulus features [20], [21], [22], [23], [24], [25]. Yet there is also evidence from a different line of research that ERP amplitudes are reduced for visual stimuli that have been repeatedly presented [26], [27], [28], [29], presumably because the neural populations processing these stimuli adapt.

If attention affects binocular rivalry by boosting neural responses to the rival stimuli, then attending to rival stimuli should *increase* ERPs from a change to a rival stimulus compared to when attention is on something else. If adaptation affects binocular rivalry and attention is accompanied by increasing adaptation, as proposed by Paffen et al., then attending to rival stimuli should *decrease* ERPs from a change to a rival stimulus. We found the latter: Attending to the rival stimuli decreases the size of the N1 compared with when attention is on something else.

## Methods

### Ethics Statement

The study was performed in accordance with the ethical standards laid down in the Declaration of Helsinki and with the ethics guidelines of the German Association of Psychology (ethics board of the Deutsche Gesellschaft für Psychologie, DGPs: http://www.dgps.de/dgps/aufgaben/ethikrl2004.pdf). Ethical approval was granted by the German Research Foundation (DFG). We obtained written informed consent from each participant.

### Participants

There were 14 participants (3 of whom were male, and 3 of whom were left handed; mean age [SD]  = 23.94 [3.9] years), all with normal or corrected-to-normal vision. The participants received either course credits or payment (6 €/hour) and were selected after they showed normal binocular rivalry in an 8-minute test session. The data of 1 participant (female, right-handed) had to be excluded from further analysis due to too many artefacts in the electrophysiological and behavioural data.

### Apparatus

During the experiment the participant sat in a sound-attenuated and electrically-shielded cabin, with his or her head stabilized by a chin rest. Participants viewed stimuli through a mirror stereoscope (Screenscope SA-200-Monitor-Type) and through a window in the cabin. The stimuli were displayed on a 19-inch, colour monitor (Llyama HM 903 DTA; 1024×768 pixels at 100 Hz). Participants responded using two buttons of a four-button response pad.

### Stimuli

The stimuli consisted of patches of black/green (CIE *x* = .282, *y* = .295, average luminance: *Y*  = 15.9 cd/m^2^, contrast = 0.78) or black/red (CIE *x* = .616, *y* = .351, *Y* = 17.7 cd/m^2^, contrast = 0.80), 1 cycle-per-degree, square-wave gratings windowed with a circular cosine envelope over 1.4° with a diameter of 5.7° of visual angle. The gratings were oriented 45° or –45° from vertical. In the middle of the stimuli was a central black fixation cross of 0.4° visual angle diameter (luminance 0.8 cd/m^2^) and which changed randomly to the letters N or Z of 0.5° visual angle. Stimuli were presented on a grey background (10.4 cd/m^2^). The horizontal positions of the stimuli and their fixation points were adjusted to allow each participant to view the two stimuli on corresponding retinal positions with normal relaxed viewing.

### Procedure

The experiment contained two different conditions. In one, we directed the participants' attention to the presented rival or fused images; we call this the *attend-to-grating condition*. In the other, we directed the participants' attention to a secondary task: they had to report changes in the fixation target while ignoring the rival or fused images; we call this the *attend-to-fixation condition*.

The basic paradigm used in both conditions was introduced by Kaernbach, Schröger, Jacobsen, and Roeber [30]. In this paradigm different periods of binocular fusion and rivalry are presented continuously in a random order (randomised afresh for each participant). We induced periods of rivalry by presenting one grating to one eye and a grating of orthogonal orientation to the other. We induced periods of fusion by presenting one grating to one eye and a grating of the same orientation to the other eye.

The continuous presentation of different rivalry and fusion periods made it possible to identify four different physical transitions: fusion–fusion and rivalry–rivalry (in which the orientation of both gratings changed in both eyes), and fusion–rivalry and rivalry–fusion (in which the orientation of only one grating changed).

Periods of rivalry stimulation lasted at least 6000 ms plus a random time between 10 ms and 2000 ms. After the end of this predefined duration a stimulus transition occurred in the attend-to-grating condition as soon as the participant pressed a key for 300 ms indicating a stable percept. To ensure that the transition was not time-locked to the key-press, we then added a random time between 10 ms and 300 ms before the transition. A stimulus transition occurred in the attend-to-fixation condition after the same duration of rivalry stimulation plus a random time of 300–1400 ms. This was to equal the typical dominance phase durations.

**Figure 1:**
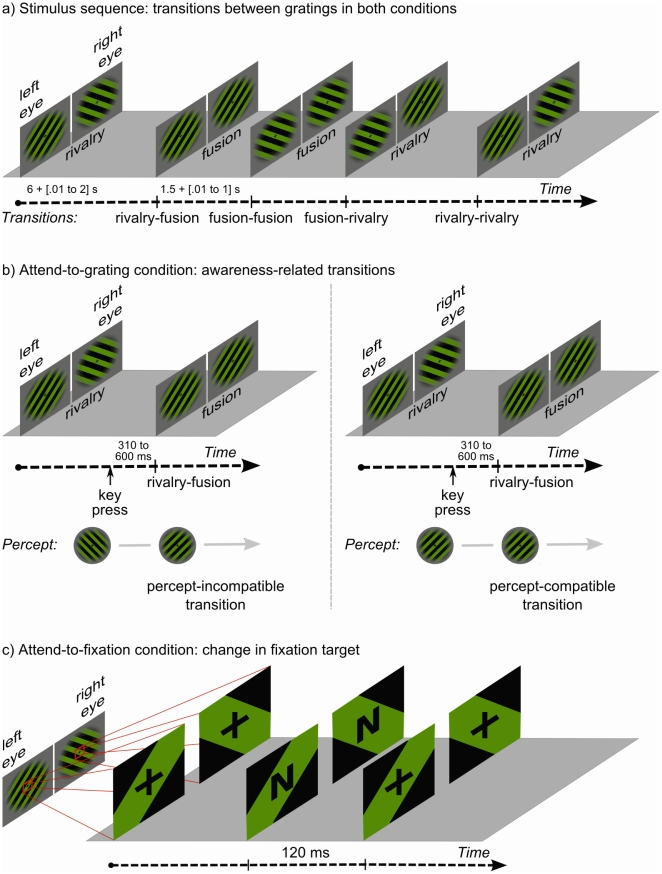
Experimental design. (a) Shows an example of the sequence of rivalry and fusion periods during the attend-to-grating and attend-to-fixation conditions. (b) Depicts the two relevant percept-dependent rivalry–fusion transitions of the attend-to-grating condition. Left: The currently perceived orientation is opposite to the change in stimulation, incompatible transitions. Right: The currently perceived orientation is the same as the change in stimulation, compatible transitions. (c) Shows an example of the fixation-cross change to one letter during a binocular rivalry period.

Periods of fusion stimulation lasted 1500 ms plus a random time between 10 ms and 1000 ms; the times of these periods were identical in the two conditions (see Figure 1a for a typical stimulation sequence).

The experiment consisted of 24 blocks of around 3 minutes each. The attend-to-grating condition and the attend-to-fixation condition alternated every fourth block and the colour of the gratings (red/black or green/black) alternated every second block. This order was counterbalanced over the participants. In the attend-to-grating condition, we asked participants to report their current perceived orientation by pressing one of two keys assigned to that specific orientation. They were to keep a particular key pressed for as long as that orientation was visible with no trace of the other and to release the key as soon as they saw any combination of the two orientations.

In the attend-to-fixation condition, the participants indicated changes of the fixation cross to either the letter N or the letter Z (Figure 1c) by pressing one of two keys. These fixation-target changes lasted 120 ms and occurred randomly between 11 to 14 times within one block. During the first 5000 ms or the last 1000 ms of a block no fixation-target change occurred. The minimum duration between two fixation-target changes was 2000 ms. The fixation-target changes were not time-locked to the stimulus transitions and served only to take attention away from rivalry or fusion stimulation.

In the attend-to-grating condition, because the participants reported their current percept, rivalry–fusion transitions can be divided into two *awareness-related* transitions (Figure 1b). When the participant saw, for example, a left-slanted grating during binocular rivalry, and stimulation changed to two, fused, right-slanted gratings, then the participant saw the orientation change. We call this a *percept-incompatible transition*. When the participant saw a right-slanted grating during binocular rivalry, and stimulation changed to two, fused, right-slanted gratings, the participant did not see the orientation change. We call this a *percept-compatible transition*.

### Electrophysiological recordings

We recorded EEG continuously with a BioSemi Activ-Two amplifier system using 128 Ag/AgCl electrodes mounted radially equidistant from Cz according to the ABC layout (http://www.biosemi.com) in an elastic cap. Additionally, we attached two electrodes to the earlobes. To monitor eye movements we recorded the horizontal and vertical electrooculograms (EOGs). The sample rate of EEG and EOGs was 512 Hz. We re-referenced the data offline to the linked earlobes. We applied 0.3–45 Hz bandpass filter (Kaiser windowed sinc FIR filter, 1857 points) to the data before analysis.

### Data analysis

We did two main sorts of analyses. The critical analyses for our purposes are for *attention-related* effects. We also analysed for *awareness-related* effects in the attend-to-grating condition, mainly to ensure that our participants confirmed what we have found earlier with similar conditions.

For attention-related analyses we compared the attend-to-grating condition with the attend-to-fixation condition. The transitions included in the analysis for the attention-related effects were all possible ones: (I) rivalry–rivalry, (II) fusion–fusion, (III) fusion–rivalry, and (IV) rivalry–fusion. These transitions allow us to compare when changes occur to one eye's stimulus (e.g., fusion–rivalry) and when changes occur to both eyes' stimuli (i.e., rivalry–rivalry). They also allow us to compare when the initial condition was rivalry and when it was fusion.

In the attend-to-grating condition we excluded all transitions from further analysis with a key press or release within 200 ms after the stimulus transition. We included transitions to fusion only for which the correct key was pressed. We included percept-compatible transitions only for which the participants continued to press a key, to make sure that the physical stimulus change was not noticed. We included all remaining rivalry-fusion transitions (i.e., percept-incompatible and percept-compatible transitions) in the attend-to-grating condition for the attention-related analyses.

In the attend-to-fixation condition, we excluded all transitions in which a fixation-target change or key press occurred between 200 ms before and 800 ms after the transitions. This was to ensure that the ERPs were not affected by activity evoked by the changes to the fixation stimulus or by activity associated with preparing a key press.

We averaged the ERP separately for the different events in a 1000 ms window, time-locked to the stimulus transitions, including a baseline from –200 to 0 ms. Prior to averaging, we rejected any transitions containing a signal change of more than 100 µV at any EOG electrode and more than 200 µV at any EEG electrode by using an automatic peak-to-peak voltage artefact detection method.

For awareness-related analyses we compared percept-incompatible transitions with percept-compatible transitions; these were possible only in the attend-to-grating condition.

We included in the attend-to-grating condition for each participant 41 (mean) ±5 (SD) rival–rival transitions, 41±4 fusion–fusion transitions, 131±16 fusion–rival transitions, and 105±15 rival–fusion transitions, separated into 61±11 percept-incompatible transitions and 44±11 percept-compatible transitions. In the attend-to-fixation condition we included for each participant 36±5 rival–rival transitions, 44±5 fusion–fusion transitions, 122±14 fusion–rival transitions, and 121±13 rival–fusion transitions.

## Results

### Behavioural data

To check that the participants focussed their attention on the fixation-target changes in the attend-to-fixation condition, we calculated the percentages of correct (i.e., the participant saw a change and identified the letter correctly), incorrect (i.e., the participant saw a change but made a mistake about what the letter was), missed responses, and false alarms. Also, we calculated the reaction times (RTs) for correct responses. The percentage of correct responses was 53±8% and the reaction time was 700±70 ms. The percentage of incorrect responses was 3±2%. The percentage of misses was 44±7%. There were only a few false alarms: six participants did not have any, the other seven had between 1% and 5% false alarms. These results indicate that the brief changes of the fixation cross to N or Z were very difficult to detect despite the participants' monitoring them very closely, showing that the attend-to-fixation task demanded a lot, if not all, of the participants' attention.

In the attend-to-grating condition, we determined the mean duration of dominance phases during rivalry stimulation. It was 1940±700 ms, which is typical for binocular rivalry with these kind of stimuli [31].

### ERP data

**Figure 2:**
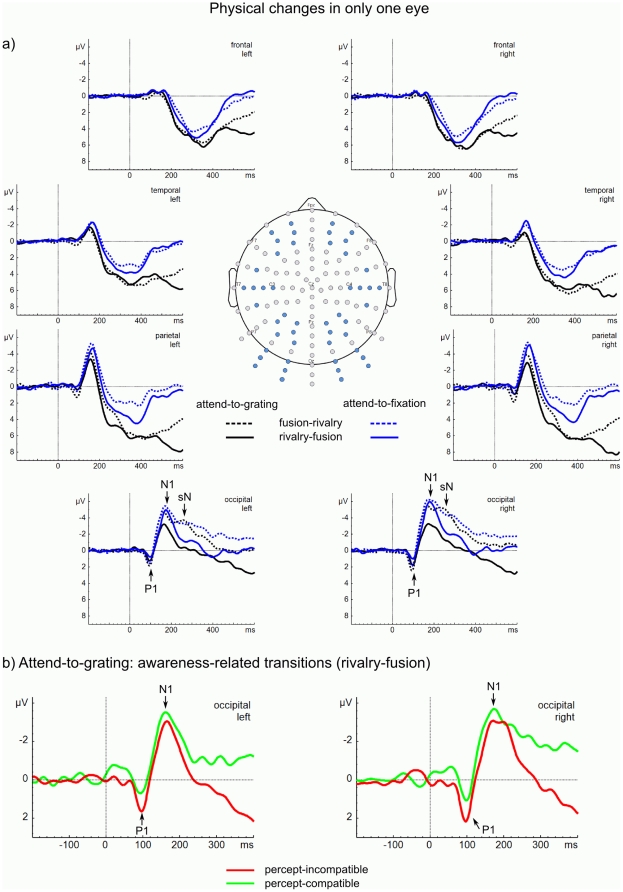
ERPs to changes in only one eye. (a) ERPs averaged within clusters of six electrodes at frontal, temporal, parietal, and occipital regions for the left and right hemispheres involving orientation changes in one eye (fusion–rivalry—dotted lines and rivalry–fusion—solid lines) from the attend-to-grating condition (black lines) and from the attend-to-fixation condition (blue lines). The major ERP components are marked with letters (P1, N1) along with a sustained negativity (sN) for fusion–rivalry. At the occipital electrodes, the N1 is greater in the attend-to-fixation conditions than in the attend-to-grating conditions. This difference persists, but is more muted, at parietal and temporal sites, and is absent at frontal sites. (b) ERPs at the occipital clusters for the awareness-related transitions of the attend-to-grating condition; incompatible (solid red line) and compatible (solid green line) transitions. The P1 is greater for percept-incompatible transitions than for percept-compatible transitions.

**Figure 3:**
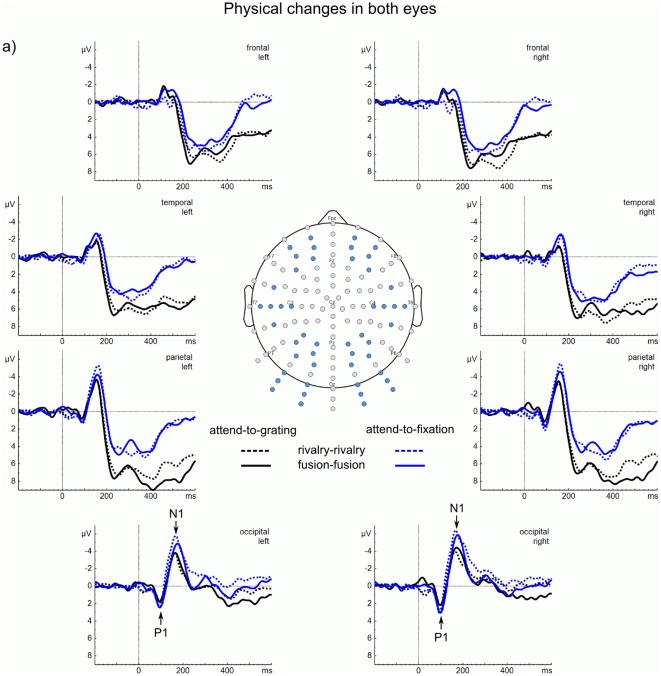
ERPs to changes in both eyes. (a) ERPs averaged within clusters of six electrodes at frontal, temporal, parietal, and occipital regions for the left and right hemispheres involving orientation changes in one eye (rivalry–rivalry—dotted lines and fusion–fusion—solid lines) from the attend-to-grating condition (black lines) and from the attend-to-fixation condition (blue lines). The major ERP components are marked with letters (P1, N1). Again, the N1 is greater in the attend-to-fixation conditions than in the attend-to-grating conditions; this is most pronounced from the occipital sites.

We show the ERP data in Figure 2 and Figure 3. To be consistent with convention, we show a plan view of a human head with the nose at the top. This means that we show ERPs from the clusters of six frontal electrodes at the top, then from six temporal electrodes, then from six parietal electrodes, and then from six occipital electrodes at the bottom of the figure. We expect the key differences in ERP components to occur in the occipital electrodes, where visual ERPs and their attentional modulation are most prominent [32] — these are the ones to look at first in the figures.

Figure 2 shows transitions in which orientation changed in only one eye (fusion–rivalry and rivalry–fusion). Figure 3 shows transitions in which orientation changed in both eyes (rivalry–rivalry and fusion–fusion). In the (a) part of each figure are the ERPs from the four regions of the left and right hemispheres. To be consistent with convention, we plotted positive deflections (P) below the Y-axis and negative deflections (N) above the Y-axis [19].

At occipital and parietal sites, all transitions elicited a P1 around 100 ms. This was followed by an N1 around 180 ms. Critically, these N1s were *smaller* in the attend-to-grating condition than in the attend-to-fixation condition. For fusion–rivalry transitions in both conditions the N1 was followed by a sustained negativity (sN) at occipital sites—a signature of rivalry (see later analysis). There were also later positive deflections around 300 ms (P3) at parietal sites for all transitions that are greater for the attend-to-grating condition than for the attend-to-fixation condition. These arise from neural activity accompanying preparation to press a key to the change in orientation that followed the transition [33]; a key press was required in the attend-to-gratings condition but not in the attend-to-fixation condition (we excluded transitions associated with a key press from the attend-to-fixation condition). Because we are mainly interested in the earliest correlates of awareness and attention we do not analyse these P3s further.

At temporal and frontal sites, all transitions elicited an N1 around 180 ms, but this was much weaker than at occipital and parietal sites, suggesting that the neural activity giving rise to the N1 is coming from the visual areas of the brain. There were also pronounced P3s that again were greater for the attend-to-grating condition than for the attend-to-fixation condition.

For all statistical analyses, we averaged ERP amplitudes for the P1 from 90 to 110 ms, for the N1 from 160 to 210 ms, and for the sustained negativity in the post-N1 interval from 210 to 300 ms at six occipital electrodes over each hemisphere. We identified these time windows by visual inspection of the grand average ERPs.

#### Attention-related results

We compared the attend-to-grating condition with the attend-to-fixation condition by using a repeated-measures ANOVA with the following factors: condition (attend-to-grating vs. attend-to-fixation), eyes' change (stimuli changed orientation in one eye vs. in both eyes), stimulation before transition (rivalry vs. fusion), and hemisphere (left vs. right).

The ANOVA for the occipital P1 showed only one significant effect: transitions that included orientation changes on both eyes elicited a larger P1 than transitions that included an orientation change on only one eye, *F*(1, 12)  = 8.12, *p*<.05, *η^2^* = .40. This is likely due to the greater change in the stimuli presented to the eyes in the former condition than in the latter; the P1 is sensitive to such physical properties of an event [34].

**Figure 4:**
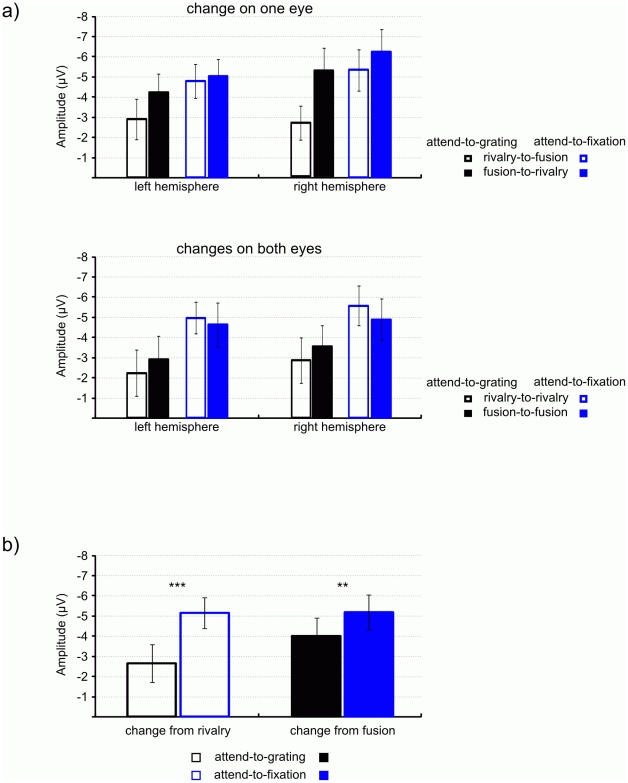
Bar graphs of the N1 averaged in the 160–210 ms time window. (a) Shows the amplitudes for transitions that involved an orientation change on only one eye (top) and for transitions that involved orientation changes on both eyes (bottom) for the attend-to-grating (black) and the attend-to-fixation (blue) conditions. Changes from rivalry are depicted as unfilled bars; changes from fusion are depicted as filled bars. (b) Plots the significant two-way interaction between condition and stimulation before transition (rivalry, fusion).

The ANOVA for the occipital N1 found a significant main effect of condition, *F*(1, 12)  = 33.78, *p*<.001, *η^2^* = .74, and of stimulation before transition, *F*(1, 12)  = 14.00, *p*<.01, *η^2^* = .54. Figure 4a depicts bar graphs of the left- and right-hemispheric occipital N1 amplitudes for all transitions in both conditions. These effects were involved in a two-way interaction, *F*(1, 12)  = 12.91, *p*<.01, *η^2^* = .52. We have plotted this interaction in Figure 4b. The figure shows (1) that the N1 is smaller in the attend-to-grating condition (achromatic bars) than in the attend-to-fixation condition (blue bars), and (2) that this difference is more pronounced for changes from rivalry, *F*(1, 12)  = 46.83, *p*<.001, *η^2^* = .80 (unfilled bars), than for changes from fusion, *F*(1, 12)  = 10.44, *p*<.01, *η^2^* = .47 (filled bars).

**Figure 5:**
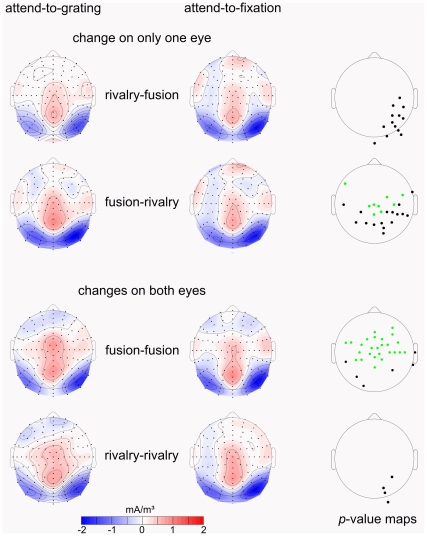
Scalp current density (SCD) maps of the N1 component from the attend-to-grating condition (left) and from the attend-to-fixation condition (middle) for transitions that involve an orientation change on only one eye (rivalry–fusion, fusion–rivalry) and for transitions that involve orientation changes on both eyes (fusion–fusion, rivalry–rivalry). The right column depicts *p*-value maps for the statistical comparison of SCD values between the attend-to-grating and the attend-to-fixation condition by means of *t*-tests. *P*-values are Bonferroni corrected for the number of electrodes (α = 0.05). Green dots mark electrodes for which the current was significantly stronger in the attend-to-grating than in the attend-to-fixation condition. Black dots mark electrodes for which the current was significantly stronger in the attend-to-fixation than in the attend-to-grating condition. Note, that at posterior electrodes for all transitions the current was stronger in the attend-to-fixation condition.

We derived the scalp current densities (SCDs) of the N1 component (Figure 5) from the ERP voltage distributions using a spherical spline surface Laplacian algorithm [35], [36]. The Laplacian was computed with the second spatial derivative of the potential distribution with a conductivity of 0.45 Siemens/m. We set the maximum degree of the Legendre polynomials to be 50 and the order of splines to be 4. For all transitions, the SCD maps show a pattern of pronounced bilateral parieto-occipital negative distributions (current sinks) and a centro-parietal positive distribution (current source). This similarity in the SCD distributions across all transitions suggests that in both tasks the same brain areas are involved in generating the N1. Hence the difference in N1 amplitude appears to be merely a quantitative difference in activation.

**Figure 6:**
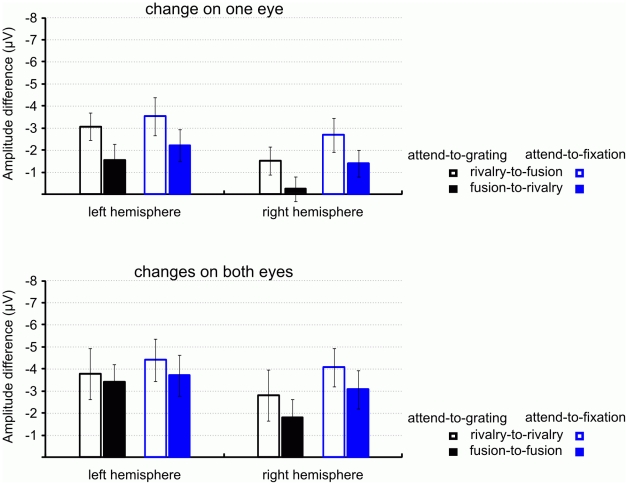
Bar graphs of the amplitude difference between N1 (160–210 ms) and post-N1 interval (210–300 ms) for transitions that involved an orientation change on only one eye (top) and transitions that involved orientation changes on both eyes (bottom) in the attend-to-grating (black) and the attend-to-fixation (blue) conditions. Changes from rivalry are depicted as unfilled bars; changes from fusion are depicted as filled bars.

When a transition that affected only one eye started with fusion (fusion–rivalry) then the ERP for the resulting rivalry continued to stay negative after the N1 (see Figure 2) but not when the transition started with rivalry (rivalry–fusion) nor when the transition affected both eyes (see Figure 2 and Figure 3). This observation was confirmed by a repeated-measure ANOVA for the differences between the N1 amplitude and the ERP amplitude in the post-N1 interval (210 ms to 300 ms). We plot the differences in ERP amplitudes as bar graphs in Figure 6. The complete ANOVA results are reported in Table 1.

**Table 1:** Results of the repeated-measures ANOVA for the differences between the N1 amplitude and the ERP amplitude in the post-N1 interval (210 ms to 300 ms).

	*F*(1,12)	*p*	*η^2^*
Condition (attend-to-grating vs. attend-to-fixation)	9.052	**.011**	**.430**
Eye's changed (change on one eye vs. changes on both eyes)	17.632	**.001**	**.595**
Stimulation before transition (rivalry vs. fusion)	2.605	.132	.178
Hemisphere (left vs. right)	5.243	**.041**	**.304**
Condition × Eye's changed	.001	.971	.000
Condition × Stimulation before transition	.066	.802	.005
Condition × Hemisphere	12.066	**.005**	**.501**
Eye's changed × Stimulation before transition	5.931	**.031**	**.331**
Eye's changed × Hemisphere	.537	.478	.043
Stimulation before transition × Hemisphere	3.923	.071	.246
Condition × Eye's changed × Stimulation before transition	.022	.886	.002
Condition × Eye's changed × Hemisphere	.470	.506	.038
Condition × Stimulation before transition × Hemisphere	.550	.473	.044
Eye's changed × Stimulation before transition × Hemisphere	.205	.659	.017
Condition × Eye's changed × Stimulation before transition × Hemisphere	.045	.836	.004

Respective follow-up ANOVAs of the two-way interaction between eyes' change (change on one eye vs. changes on both eyes) and stimulation before transition (rivalry vs. fusion) revealed that when the physical change occurred on only one eye the post-N1 amplitude stayed more negative for transitions starting from fusion (fusion–rivalry) than for transitions starting from rivalry (rivalry–fusion), *F*(1, 12)  = 13.43, *p*<.01, *η^2^* = .53. When the physical changes occurred on both eyes fusion–fusion and rivalry–rivalry transitions showed about the same amplitude decrease in the post-N1 interval: *F*(1, 12)  = 1.89, *p*>.1, *η^2^* = .14.

In summary, the attention-related results show that the earliest effects of attention on rivalry emerge in the N1 component of the ERPs. Critically, attending to the rivalry stimuli yields smaller N1s than when attention is on the fixation stimuli. That rivalry stimuli are processed differently from fusion stimuli independent of the attention devoted to them is shown by the sustained negativity in the post-N1 interval for fusion–rivalry transitions.

#### Awareness-related results

In the (b) part of Figure 2, we give an expanded view of the occipital ERPs for the left and right hemispheres elicited by percept-incompatible and percept-compatible rivalry–fusion transitions in the attend-to-grating condition. To investigate the earliest awareness-related effect in the attend-to-grating condition we compared percept-incompatible and percept-compatible transitions. For the P1, we calculated a repeated-measures analysis of variance (ANOVA) including two factors: transition (incompatible vs. compatible) and hemisphere (left vs. right) for the occipital electrode clusters. This showed that the P1 was larger for incompatible transitions than for compatible transitions in both hemispheres, *F*(1, 12)  = 11.08, *p*<.01, *η^2^* = .48. This is consistent with earlier work [31], [37], [38].

We could not investigate the earliest awareness-related effect in the attend-to-fixation condition because the participants did not report their perceived orientations.

## Discussion

Our primary goal was to investigate what happens in the brain when attention is on binocular rivalry stimuli compared with when attention is on something other than the rival stimuli. We found that the N1 (160–210 ms) is *smaller* when attention is on the rival stimuli compared with when attention is diverted from the rival stimuli. We propose that this N1 effect is from differential adaptation to the rivalry stimuli in the two conditions. Our secondary goal was to confirm that visual consciousness affects the size of the P1. We discuss the second goal first, because it bears on the sensitivity of our data to uncover subtle effects.

### Awareness-related effects

We found the earliest neural activity correlated with visual consciousness in the P1-range after 100 ms in the attend-to-grating condition. This effect has been found with the same stimuli [31] and with different stimuli using the same paradigm [37], [38] as well as in other experimental paradigms [39], [40], [41], [42]. Similar awareness-related effects around 100 ms have been found for binocular rivalry using a different approach [43] and for Necker cube reversals [40].

### Attention-related effects

We address two key questions:

*Could our attention effects have come because attending to the fixation stimuli essentially abolished rivalry?* Having no rivalry when attention is diverted from the rival stimuli seems—at a first glance—a possible explanation for our results. It would also be consistent with the slowing of rivalry with diverted attention Paffen et al. [14] observed and with the results of Zhang et al. [16]. But we can dismiss this explanation for at least three reasons.

First, O'Shea [44] took attention and responses away from rival gratings by asking his participants to respond to onset of a small, central, monocular, fixation point. He found that there were some extremely slow reaction times, of the order of several seconds, that did not occur when the stimuli were fused. He concluded that rivalry from the ignored gratings had suppressed visibility of the point, suggesting that rivalry continues without attention.

Second, Pastukhov and Braun [45] had their observers track rivalry (admittedly not binocular rivalry) in the usual way, but inserted varying periods of time during which attention was diverted to a very difficult peripheral task. They were able to calculate the rate of rivalry during these times by measuring the proportion of trials during which perception changed from before the period to after. If rivalry was abolished during these periods, then perception should never have changed. But it did, albeit at a slower rate, also suggesting that rivalry continues without attention.

Third, as an indirect measure from our own data, the ERPs elicited by the fusion–rivalry transitions show a sustained negativity, characteristic of processing dichoptic stimulation, around 210–300 ms after rivalry onset. This dichoptic stimulation–specific sustained negativity occurs regardless of whether attention was on the gratings or not (Figure 2 and Figure 6), suggesting that rivalry might continue without attention.

In sum, we compared the processing of congruent and incongruent binocular stimuli, when they were either attended (task-relevant) or not attended (task-irrelevant). We find processing differences between congruent and incongruent binocular stimuli, when they were attended (attend-to-grating condition). Hence these differences are related to perception. We find rather similar differences (in terms of timing, morphology and topography) when the stimuli were not attended (attend-to-fixation condition). Although we cannot link these differences directly to perception or fluctuations in perception, they reveal a differential processing of rival and non-rival stimuli. Finding the same differences in both conditions suggests that the same distinction between rival and non-rival stimuli is made by the neural mechanisms processing the stimuli irrespective of the stimuli's task-relevance.

*Why did we get smaller ERPs with attention whereas other researchers got larger ERPs?* There are numerous procedural differences between our experiment and those showing enhanced ERPs from attention during binocular rivalry [17], [18]. But if we are right about adaptation being the key underlying process [46], then there are two critical aspects: First, the event needs to be processed by an adapted mechanism. This is certainly true in our experiment: during rivalry attention was on both orthogonal rival gratings, leading to adaptation of mechanisms processing both orientations. And the event involved changing one rival stimulus to be the same as the other, ensuring that the event was processed by adapted mechanisms.

Second, the event needs to occur after a long enough time for attention to result in significant adaptation. Events that occur shortly after attention is brought to bear on a stimulus, before much adaptation has accrued, will result in an enhanced ERP. Studies showing such enhanced ERPs in rivalry [17], [18] had events that occurred within about 400 to 800 ms of the allocation of attention. Whereas events that occur a long time after attention is brought to bear on a stimulus, after a lot of adaptation has accrued, will result in a reduced ERP. In our study, voluntary attention needed to be sustained on the rival stimuli for up to eight seconds before an event.

There is at least one previous report by Rugg, Milner, Lines, and Phalp, in a task other than rivalry, that the N1 (N180) elicited in an attend-to-stimulus condition is smaller than the N1 in an unattended condition [47]. Rugg et al. describe their finding as “[a] puzzling feature of the data … clearly in need of replication” (p. 94). We are not aware of any study that followed up on this report. But we think that Rugg et al.'s finding is consistent with adaptation.

On any trial, Rugg et al. showed participants a single white bar on a black background. The bar could be either horizontal or vertical. On most trials, the bar was thick; occasionally it was slightly thinner. The participants' task in a block of trials was to press a key for, say a thin horizontal bar. Rugg et al. compared ERPs to thick horizontal bars in blocks of trials when participants were looking for thin horizontal bars (attend condition) with ERPs to thick horizontal bars in blocks of trials when participants were looking for thin vertical bars (unattend condition). Critically, these blocks were three minutes long, comprising 100 trials, during which participants had to hold their attention.

We propose that the smaller N1 Rugg et al. found in the attend conditions was because the sustained attention on one particular orientation increased adaptation for that orientation. This means that when the critical stimulus was shown, it was processed by an adapted population of neurons, leading to less activity. If we are right about this, adaptation not only explains our results, but also resolves a long-standing mystery in the literature.

### Summary and conclusions

First, we confirmed that the first awareness-related modulation following binocular rivalry is in the P1 (90–110 ms) [31], [37], [38], [42]. Second, the prolonged negativity following N1 for fusion–rivalry but not for rivalry–fusion stimuli observed in both conditions suggests that binocular rivalry might take place when attention is diverted from the rivalry stimuli. Third and critically, we found that the N1 (160–210 ms) is smaller when attention is on the rival stimuli then when attention is diverted from the rival stimuli.

We conclude that the N1 effect very likely is from differential adaptation to the rivalry stimuli in the two conditions. Our results provide evidence that binocular rivalry processing is affected by attention but cannot be fully explained by it.
